# Both air-sea components are crucial for El Niño forecast from boreal spring

**DOI:** 10.1038/s41598-018-28964-z

**Published:** 2018-07-12

**Authors:** Xiang-Hui Fang, Mu Mu

**Affiliations:** Department of Atmospheric and Oceanic Sciences & Institute of Atmospheric Sciences, Fudan University, Shanghai, China

## Abstract

The spring predictability barrier severely limits our ability to forecast the El Niño-Southern Oscillation (ENSO) from and across the boreal spring. Our observational analysis shows that the spring predictability barrier (SPB) can be largely reduced when information from both the ocean and atmosphere are effectively taken into account during the boreal spring. The correlation coefficient between the predicted and observed sea surface temperature anomalies over the equatorial central–eastern Pacific determined by a simple quaternary linear regression model is >0.81 for the period 1980–2016. The frame structure of the ENSO evolution is mostly controlled by variations in the oceanic heat content along the equatorial Pacific and the zonal wind stress over the tropical western Pacific during the boreal spring. These results indicate that to predict ENSO events with a long lead time, i.e., largely reducing the SPB, variations in both the ocean and atmosphere during the boreal spring should be well predicted first. While the oceanic information is mainly located in the equatorial Pacific and well characterized by the delayed oscillator and recharging oscillator models, variations in the atmosphere may contain information beyond this area and are more difficult to deal with.

## Introduction

As the dominant interannual variability, El Niño-Southern Oscillation (ENSO) has far-reaching impacts on climate and society around the world^1^. The simulation and forecasting of ENSO is therefore of great importance. As the leading paradigm for the ENSO theory, the recharge oscillator emphasizes the time delay between anomalies in longitudinally averaged thermocline depth (TCD; approximated by the depth of the 20 °C isotherm) and the eastern Pacific (EP) sea surface temperature (SST) and explains the periodic characteristics of ENSO^2,3^. Analyses of observational data have confirmed this paradigm and have suggested that the oceanic heat content along the equatorial Pacific is a good predictor of the ENSO events^4^. Recent work has indicated that if the changing background state is accounted for, all flavours of El Niño will have similar early subsurface origins^5^. These results emphasize the important part played by the ocean in the ENSO system and suggest that it may be possible to predict ENSO events with a long lead time. However, forecasts of ENSO are limited by the spring predictability barrier (SPB), which refers to a sudden reduction in the ENSO forecast skills across the spring period in most climate models^4,6–9^. As indicated in ref.^10^, the skill of model runs based on observations in the boreal spring to predict the boreal winter SST anomalies averaged over the Niño3.4 region (170° W–120° W, 5° S–5° N) is poor, with a correlation coefficient of about 0.6 between the predicted and observed values.

In fact, in addition to the subsurface ocean heat condition, the atmosphere also plays an important role in forcing the ENSO system. For example, the westerly wind burst, an intraseasonal phenomenon occurring in the equatorial western and central Pacific, is widely believed to be an important trigger for El Niño events^11–15^. Based on numerical experiments, it has been shown that, with the same initial ocean heat content along the equatorial Pacific, an additional westerly wind burst during the boreal spring could change an original neutral event into an El Niño event^15^. This suggests that we need to extract the exact information from both the ocean and the atmosphere during the boreal spring to be able to predict the ENSO events and reduce the SPB. We show here that it is possible to successfully predict ENSO starting from the boreal spring using a simple linear regression method that considers the variations in both air–sea components.

The first predictor for the ENSO system is the oceanic heat content along the equatorial Pacific, which is defined here as the basin mean (120° E–80° W, 2° S–2° N) TCD anomalies (TCDa_M hereinafter). According to the recharge paradigm, the progress of the oceanic heat content from accumulation to release (i.e., discharge) is important in the development of El Niño, and the recharge progress (i.e., from release to accumulation) is important in the development of La Niña^5,16,17^. However, we cannot give an accurate prediction of the ENSO during the boreal spring based on this information alone. The correlation coefficient between the TCDa_M index during the boreal spring (March–May) and the Niño3.4 SST anomaly index during the boreal winter (October–December) is only 0.56 for the period 1980–2016 (see Figure S1 in the supplementary information). Although passing the 95% confidence level using Student’s *t*-test with 18 degrees of freedom, this relatively low value indicates that the discharging (recharging) process during the boreal spring is not a sufficient condition for leading the occurrence of El Niño (La Niña) events. Information from the atmosphere should also be considered.

**Figure 1:**
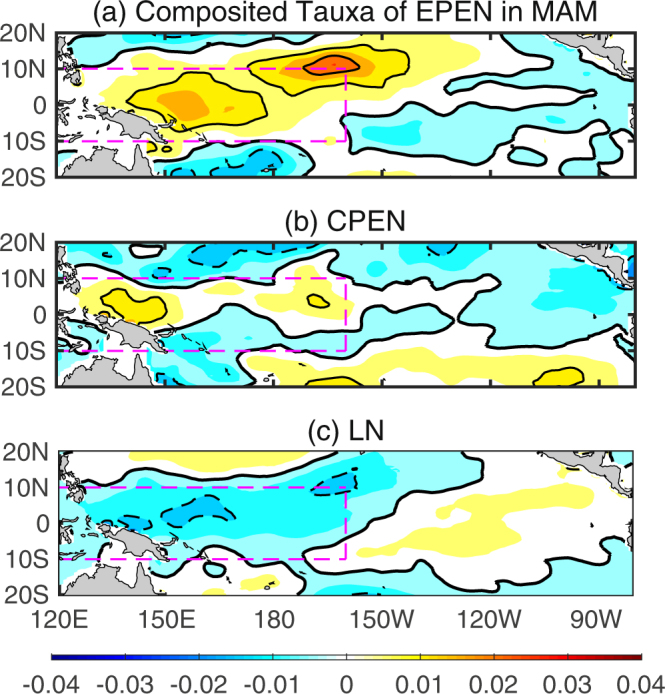
Composite mean Tauxa of the ENSO events during the boreal spring (March–May). (**a**) EP El Niño. (**b**) CP El Niño. (**c**) La Niña. The contour interval is 0.01 N/m^2^. The purple box represents the region (120° E–160° W, 10° S–10° N).

Figure 1 shows the composite mean of the anomalous zonal wind stress (Tauxa) of the EP El Niño (the canonical El Niño with the maximum SST anomalies located in the EP), central Pacific (CP) El Niño (the new type of El Niño with maximum warming anomalies confined mostly in the CP) and La Niña events during the boreal spring. Although different types of ENSO make the distribution of SST anomalies complex, the westerly (easterly) Tauxa during El Niño (La Niña) events are concentrated in similar regions over the tropical western Pacific (the purple box in Fig. 1). Physically, these anomalous zonal wind stresses could stimulate Kelvin waves on the oceanic TCD and the thermally coupled Walker mode on the oceanic surface to trigger the ENSO event^18–22^. The thermally coupled Walker mode is used by ref.^18^ to refer the Southern Oscillation mode of variability that emerges from the thermodynamic coupling to an ocean mixed layer, i.e., without dynamical coupling to the ocean. The high correlation coefficient between the box mean Tauxa (Tauxa_W hereinafter) during the boreal spring and the Niño3.4 SST anomaly index during the boreal winter is 0.71 for the period 1980–2016 and passes the 95% confidence level using Student’s *t*-test with 19 degrees of freedom, confirming the important role played by the atmosphere. This also indicates that the Tauxa_W index during the boreal spring could be another good predictor of the ENSO events. It is encouraging that the TCDa_M and Tauxa_W indices during the boreal spring are linearly independent, i.e., their correlation coefficient during 1980–2016 is only 0.15. As a result, we believe that a bivariate linear regression model using the TCDa_M and Tauxa_W indices during the boreal spring could give a more accurate prediction of ENSO for the following seasons than a model that relies on any one predictor.

Based on this simple model, the predicted SST anomalies during the boreal winter over the equatorial central–eastern Pacific are similar to the observations for the period 1980–2016, i.e., the correlation coefficients are 0.84, 0.85 and 0.71 for the Niño3.4, Niño3 (150° W–90° W, 5° S–5° N) and Niño4 (160° E–150° W, 5° S–5° N) indices, respectively. They all pass the 95% confidence level using Student’s *t*-test. Meanwhile, the regression coefficients of the TCDa_M and Tauxa_W indices are 0.55 ± 0.23 and 0.76 ± 0.23, respectively. They represent the relative contribution of the ocean and atmosphere to the evolution of ENSO and are denoted by 95% confidence intervals using Student’s *t*-test. It indicates that the combined role of the oceanic and atmospheric parts during the boreal spring is required to induce an ENSO event and ignoring either one of these parts will severely reduce the prediction skills of the model.

**Figure 2:**
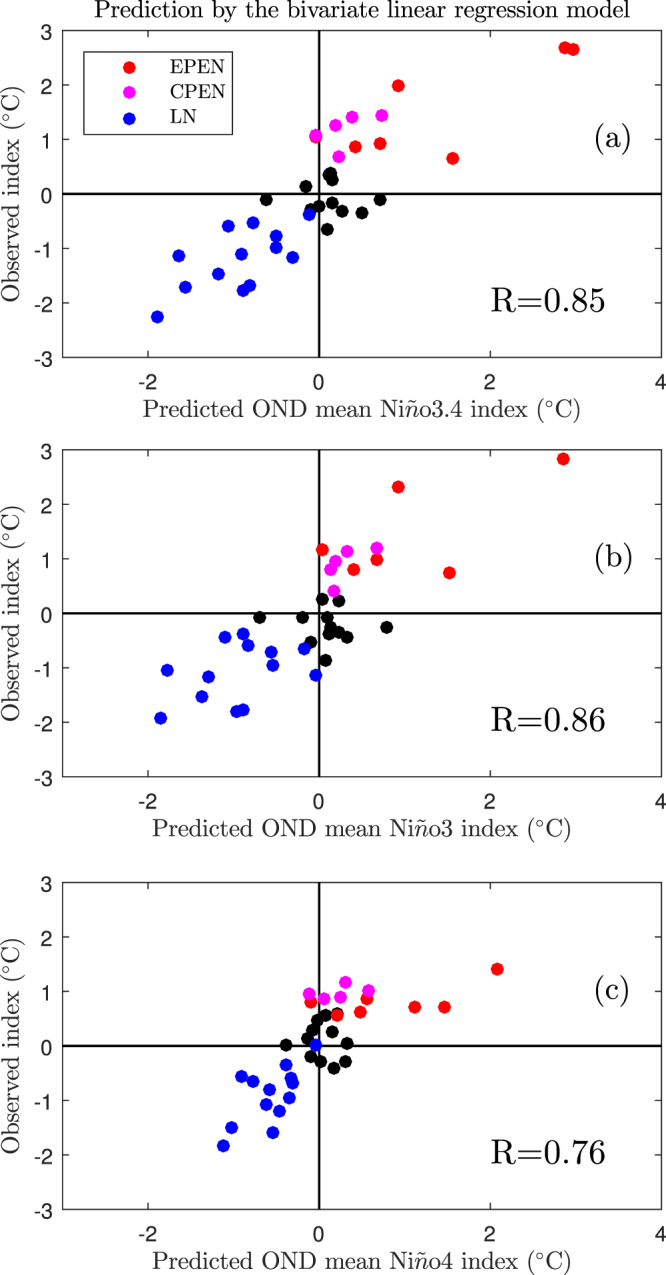
Relations between the predicted and observed Niño indices during the boreal winter. The predicted indices are obtained by the bivariate linear regression method using the TCDa_M and Tauxa_W indices during the boreal spring as the predictors. Panels (a–c) are for the Niño3.4, Niño3 and Niño4 indices, respectively. In each panel, the red, purple and blue dots are for the eastern Pacific El Niño, the central Pacific El Niño and La Niña events, respectively, whereas the black dots are for neutral years. The correlation coefficients between the predicted and observed Niño indices are also shown in each panel. OND represents the boreal winter season, i.e., October, November and December.

Considering the role of the TCDa_M index in predicting ENSO decreased suddenly from 1980–1999 to 2000–2016^23,24^, i.e., a decadal variation, the bivariate linear regression models are therefore constructed separately for these two periods. Then these two models are used to predict the following evolution of ENSO during 1980–1999 and 2000–2016, respectively. Figure 2 shows the total predicted results, i.e., 1980–2016. The correlation coefficients between the predicted and observed Niño3.4, Niño3 and Niño4 indices during the boreal winter are as high as 0.85, 0.86 and 0.76, respectively. Besides, apart from the two El Niño events, i.e., 1987–1988 and 1994–1995, all the ENSO types are successfully predicted during the boreal spring, i.e., El Niño and La Niña events are located in the first and third quadrants, respectively. The change in the regression coefficients also shows that although the contribution from the TCDa_M (0.64 ± 0.40) and Tauxa_W (0.59 ± 0.40) indices are almost equivalent during the first period, the Tauxa_W index (0.95 ± 0.33) plays a dominant role over the TCDa_M index (0.47 ± 0.33) in predicting the ENSO events during the more recent period. This indicates that more information about the ENSO system is contained in the atmosphere than in the ocean during the boreal spring in the 2000–2016 period. On the one hand, these changes are consistent with the argument from ref.^23^ that the role of the TCDa_M index in predicting ENSO decreased suddenly from 1980–1999 to 2000–2016. On the other hand, they are consistent with the fact that the central Pacific type of El Niño occurred more frequently during the recent decade, for which the dominant dynamic processes are the zonal advective feedback and the latent heat feedback^25,26^, which are both related with the zonal wind anomalies over the western and central Pacific region.

**Figure 3:**
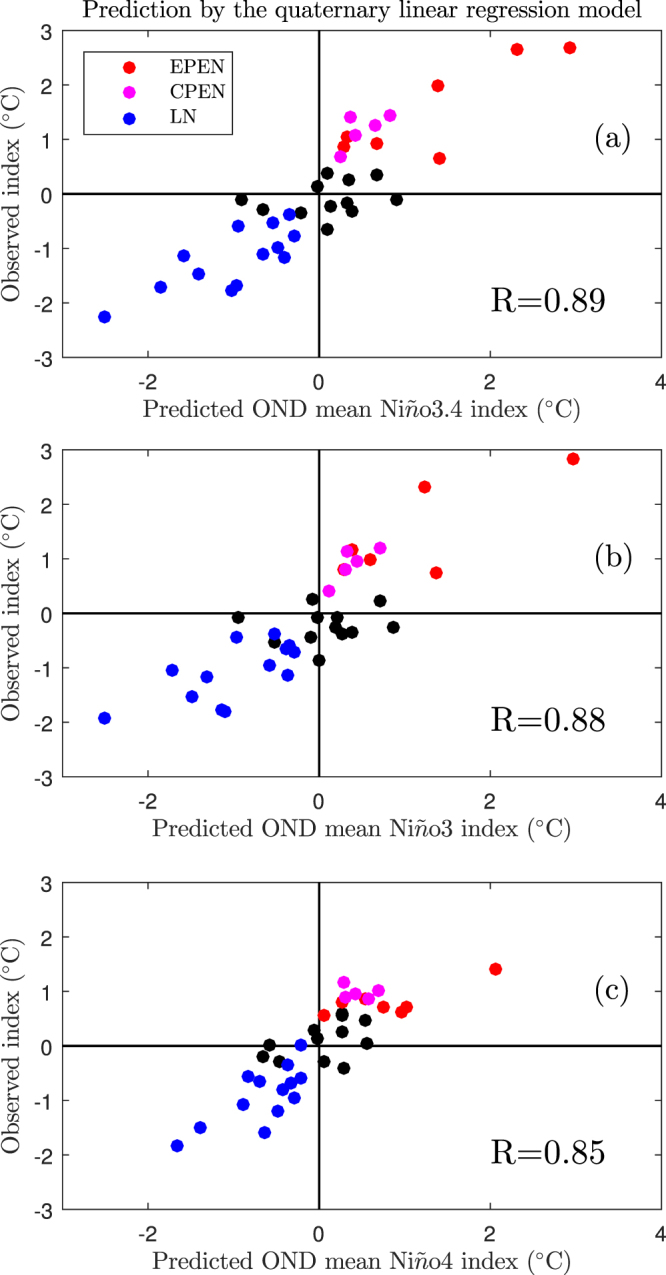
Relations between the predicted and observed Niño indices during the boreal winter. The predicted indices are obtained by the quaternary linear regression method that using the TCDa_M, Tauxa_W, central Pacific and eastern Pacific indices during the boreal spring. Panels (a–c) are for the Niño3.4, Niño3 and Niño4 indices, respectively. In each panel, the red, purple and blue dots are for the eastern Pacific El Niño, the central Pacific El Niño and La Niña events, respectively, whereas the black dots are for neutral years. The correlation coefficients between the predicted and observed Niño indices are also shown in each panel. OND represents the boreal winter season, i.e., October, November and December.

Since the concept of two types of El Niño event first emerged^27–31^, the complexity and uncertainty of ENSO have increased^32–34^. To utilize the SST information and take the different types of ENSO into account, we further add the CP and EP indices^28^ into the predictors during the boreal spring, i.e., we construct a quaternary linear regression model to predict the boreal winter El Niño indices. Again, the separate models are constructed for the periods 1980–1999 and 2000–2016, respectively, and these two models are used to predict the following evolution of ENSO during their corresponding periods. The total predicted results (i.e., 1980–2016) are shown in Fig. 3. We obtain an improved prediction, especially for the Niño4 index, when the correlation coefficient between the prediction and observations is as high as 0.85. All the different types of ENSO are now successfully predicted during the boreal spring. This means that by considering the SST of the two types of ENSO events during the boreal spring, the SST anomalies over the equatorial central–eastern Pacific region during the boreal winter can be predicted successfully.

The contribution rates of the four indices during the two periods show that the dominant predictors are still the TCDa_M index during 1980–1999 and the Tauxa_W index during 2000–2016. Taking the Niño3.4 index prediction as an example, the regression coefficients of the TCDa_M, Tauxa_W, CP and EP indices during the period 1980–1999 are 0.98 ± 0.46, 0.25 ± 0.43, 0.65 ± 0.49 and 0.54 ± 0.51, respectively. During the period 2000–2016, these regression coefficients are 0.36 ± 0.46, 1.05 ± 0.43, −0.14 ± 0.76 and 0.1 ± 0.65, respectively. This confirms that the main frame structure of the ENSO system could be mostly controlled by variations in the oceanic heat content along the equatorial Pacific and the zonal wind stress over the tropical western Pacific and that their relative roles have significant decadal variations.

**Figure 4:**
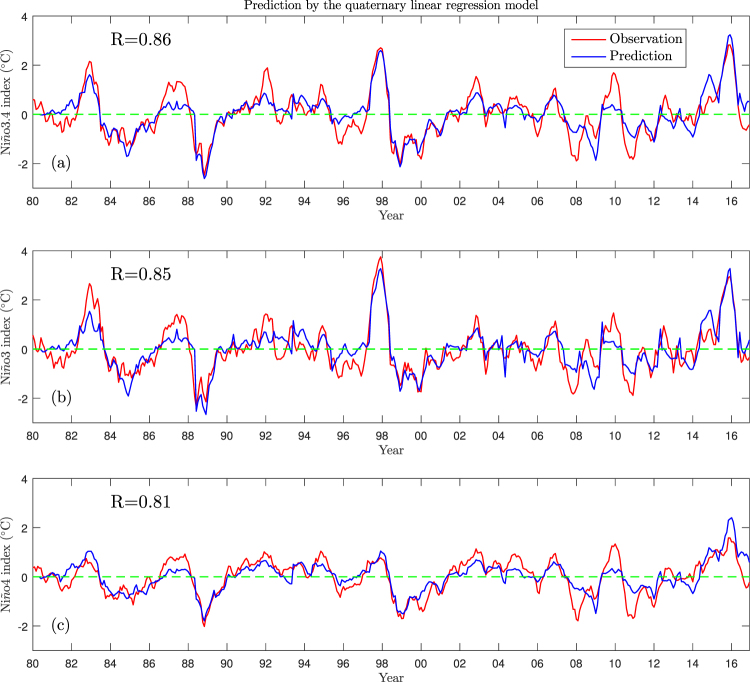
Retrospective predictions of the El Niño indices in each month from June 1980 to December 2016. Panels (a–c) are for the Niño3.4, Niño3 and Niño4 indices, respectively. In each panel, the red lines are for the observed indices and the blue lines are the predictions (from June to the following May of each year) using the quaternary linear regression method using the TCDa_M, Tauxa_W, central Pacific and eastern Pacific indices during the boreal spring. The correlation coefficients between the predicted and observed El Niño indices are also shown in each panel.

Using the quaternary linear regression method, the SST anomalies from June to the following May of each year can all be predicted based on information from the boreal spring. Figure 4 shows the predicted and observed El Niño indices of all the months from June 1980 to December 2016. The predictions are quite similar to the observations, except for the prediction of the intensity of ENSO in some years—for example, the predicted 2007–2008 and 2010–2011 La Niña and the 1986–1988 and 2009–2010 El Niño events are too weak, whereas the 2014–2015 El Niño event is too strong. The correlation coefficients of the Niño3.4, Niño3 and Niño4 indices in the period 1980–2016 are as high as 0.86, 0.85 and 0.81, respectively. Thus if we take the information from both the ocean and atmosphere during the boreal spring into right consideration, the subsequent evolution of air–sea interactions over the equatorial Pacific (the ENSO events) can be largely predicted.

In summary, although remarkable achievements have been made in the simulation and prediction of the ENSO events, the skill for predicting ENSO starting from the boreal spring is still poor, making predictions from a long lead time difficult. Because the memory of the ocean is much longer than that of the atmosphere, the prediction of the ENSO events is considered to be dominated by oceanic processes. This has led to the classic ENSO paradigms—the delayed oscillator^35,36^ and the recharging oscillator—that consider the ENSO phenomenon as a slightly damped periodic oscillation modulated by stochastic noise^37–39^. However, recent work has found that, with the same distribution of the oceanic subsurface temperature, an additional westerly wind burst during the boreal spring could change the original neutral event into an El Niño event. This suggests that the ENSO system starting in the boreal spring does not only rely on the oceanic heat content and that exact information from the atmosphere is also crucial. This has been especially true during recent decades, when the part played by the oceanic heat content in predicting the ENSO events has shown a sudden decrease.

We extracted information from both the ocean and atmosphere during the boreal spring, i.e., the TCDa_M index that representing the oceanic heat content, the Tauxa_W index that representing the atmospheric forcing, the CP and EP indices that representing the SST distribution for the two types of ENSO events. We then used a quaternary linear regression method to predict the subsequent SST anomalies over the equatorial Pacific from June to the following May. The correlation coefficients between the predicted and observed SST anomalies over the equatorial central–eastern Pacific were >0.81 for the period 1980–2016 and all the different types of ENSO were successfully predicted during the boreal spring. The success of the retrospective prediction relies mainly on the TCDa_M and Tauxa_W indices and their relative contributions have significant decadal variations. It is interesting to note that a similar successful statistical model was also constructed recently to overcome the SPB issue of the ENSO forecast^40^. Different from the two predictors used in our work, the oceanic predictor in their model is the potential temperature anomalies over the 130° E–180°, 5° N–5° S, 5–250 m area and the atmospheric predictor is the cumulative zonal wind anomalies over the 140° E–160° W, 5° N–5° S area and integrated from November up to the prediction month. But consistently, the two successful models both deeply rely on the information from both the ocean and atmosphere during the boreal spring.

These results indicate that to predict the ENSO events with a longer lead time, i.e., largely reducing the SPB, variations in both the ocean and atmosphere during the boreal spring should be well predicted first. It should be emphasized that the TCDa_M and Tauxa_W indices used in this article represent different mechanisms for the evolution of the ENSO system. Specifically, the TCDa_M index mainly reflects the classic ENSO perspective (e.g., the delayed oscillator and recharging oscillator), which treat the ENSO system as a linear oscillating phenomenon based on the wave dynamics and the water exchange between the equatorial and off-equatorial regions, respectively. While the Tauxa_W index in the boreal spring (the weakest air-sea interaction over the equatorial Pacific during a year), which can stimulate Kelvin waves on the oceanic TCD and the thermally coupled Walker mode on the oceanic surface to influence the ENSO evolution, mainly reflects the influences beyond the linear oscillating framework. This includes the signals from the westerly wind burst (WWB)^12,14^, signals from the northeast^41–45^ and southeast Pacific^46^ through the seasonal footprinting mechanism and signals from the Indian Ocean^47^ and Atlantic Ocean^48–51^. through anomalous atmospheric circulation and waves, etc. The decadal variations of the relative contributions of the TCDa_M and Tauxa_W indices indicate the different importance of their related physical processes, e.g., the influences beyond the linear oscillating framework is more important in the recent decade for reducing the SPB. In addition, the main periods of the TCDa_M and Tauxa_W indices are 3.7 and 2.5 years, respectively (figures not shown). This difference gives us a clue to investigate the essence of the wide range of the ENSO irregular period (generally 2–7 years)^38^, and also the decadal variations of the ENSO variability and frequencies^52,53^.

## Methods

### Datasets

The observational and reanalysis datasets were obtained from the National Centers for Environmental Prediction Global Ocean Data Assimilation System (GODAS)^54^. The datasets were analysed for the period 1980–2016 and the mean seasonal cycles were removed to calculate the anomalies in each field. The equatorial TCD was defined as the depth at which the oceanic potential temperature was 20 °C. The TCDa_M index, which represents the oceanic heat content in the equatorial Pacific, was calculated as the area mean of the TCD anomalies in the region (120° E–80° W, 2° S–2° N). The Tauxa_W index was calculated by the area mean of Tauxa over the region (120° E–160° W, 10° S–10° N). The CP and EP indices were calculated based on the regression-empirical orthogonal function method introduced in ref.^27^. The TCDa_M, Tauxa_W, CP and EP indices were all standardized before they were used to construct the linear regression model.

### Bivariate linear regression model

To predict the winter El Niño indices based on the air–sea information during the boreal spring, we first constructed a bivariate linear regression model. The model is as follows:

1 $$Nin{o}_{p}^{OND}=a\times TCDa\_{M}_{o}^{MAM}+b\times Tauxa\_{W}_{o}^{MAM}+c$$

where $Nin{o}_{p}^{OND}$ is the predicted El Niño index in the boreal winter (October-December) and $TCDa\_{M}_{o}^{MAM}$ and $Tauxa\_{W}_{o}^{MAM}\,\,$are the observed TCDa_M and Tauxa_W indices during the boreal spring (March-May), respectively. *a*, *b* and *c* are the regression coefficients and are obtained by replacing $Nin{o}_{p}^{OND}$ with the observed Niño index in the boreal winter. This regression method was used to predict all the El Niño indices separately.

### Quaternary linear regression model

This is similar to the bivariate linear regression model, but with the addition of the CP and EP indices during the boreal spring:

2 $$Nin{o}_{p}^{OND}=a\times TCDa\_{M}_{o}^{MAM}+b\times Tauxa\_{W}_{o}^{MAM}+c\times C{P}_{o}^{MAM}+d\times E{P}_{o}^{MAM}+e$$

where $C{P}_{o}^{MAM}$ and $E{P}_{o}^{MAM}\,$are the observed CP and EP indices during the boreal spring, respectively. *a*, *b*, *c*, *d* and *e* are the regression coefficients obtained by replacing $Nin{o}_{p}^{OND}$ with the observed Niño index in the boreal winter. The model used to predict the SST anomalies over the equatorial Pacific from June to the following May during each year was constructed using a similar method with the term on the left-hand side replaced by the SST anomalies in each month.

### Statistical analysis

Because the data from the geophysical time series are not always independent of each other, the persistence and the finite size of the samples must be considered when computing the statistics^55,56^. In this method, the degrees of freedom can be derived for significance tests using the correlation between two time series (*X*_*i*_ and *Y*_*i*_) with different autocorrelation sequences of ${\rho }_{\tau }^{X}$ and ${\rho }_{\tau }^{Y}$ as follows^56^:

3 $${T}_{XY}^{\ast }=\frac{T}{{\sum }_{\tau =-(T-1)}^{(T-1)}(1-|\tau |/T){\rho }_{\tau }^{X}{\rho }_{\tau }^{Y}}$$

where ${T}_{XY}^{\ast }$ is the number of degrees of freedom used in the significance calculation and *T* is the unadjusted number of degrees of freedom.

## Electronic supplementary material

Supplementary Information
