# Supplementary material for: Both air-sea components are crucial for El Niño forecast from boreal spring
Source: Sci Rep. 2018 Jul 12;8:10501. doi: 10.1038/s41598-018-28964-z (PMC6043610; doi:10.1038/s41598-018-28964-z)
Supplement: Supplementary file 1 — Supplementary Information [file 41598_2018_28964_MOESM1_ESM.pdf]

Supplementary Information

# Both air-sea components are crucial for El Niño forecast from boreal spring

Xiang-Hui FANG[1] and Mu MU[1]

[1.] Department of Atmospheric and Oceanic Sciences & Institute of Atmospheric Sciences, Fudan University, Shanghai, China

* *Corresponding author*: Dr. Xiang-Hui Fang
Email: fangxh@fudan.edu.cn

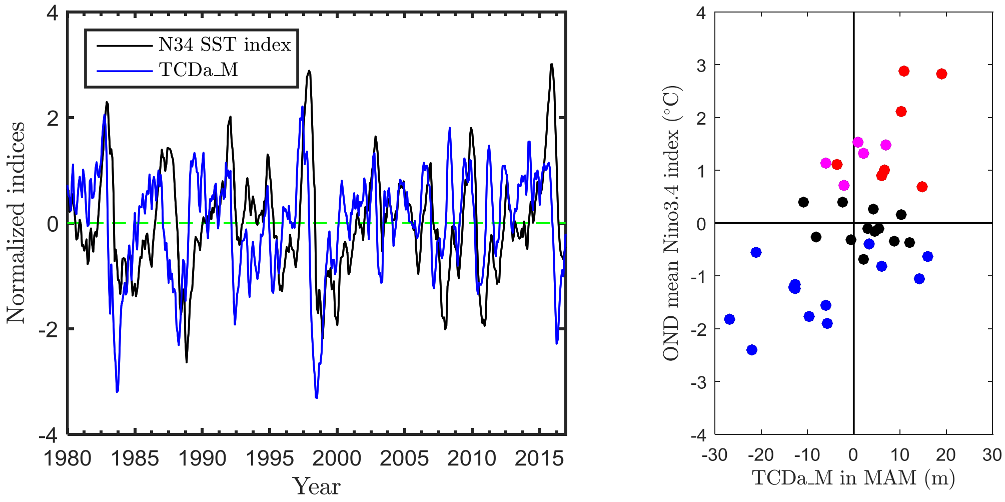

Figure S1. Relations between the TCDa_M and Niño3.4 SST index. Left panel is the monthly variations of the normalized TCDa_M index (blue), which is defined as the basin mean (120° E–80° W, 2° S–2° N) TCD (thermocline depth) anomalies, and the normalized Niño3.4 SST (sea surface temperature) index (black). Right panel shows the March-April-May (MAM) mean TCDa_M index versus the October-November-December (OND) mean Niño3.4 SST index. In the panel, the red, purple and blue dots are for the eastern Pacific El Niño, the central Pacific El Niño and La Niña events, respectively, whereas the black dots are for neutral years. It should be noted that the TCDa_M index is nearly identical with the frequently used WWV (warm water volume) index, which is defined as the basin mean TCD anomalies in the region (120° E–80° W, 5° S–5° N). We choose the TCDa_M index as the predictor because the correlation coefficient between the OND mean Niño3.4 index and the MAM mean TCDa_M index (0.56) is a little higher than that with the MAM mean WWV index (0.55) and TCDa_M is more concentrated to the equator to reflect the equatorial dynamics.
